# Mapping neuronal inputs to *Kiss1* neurons in the arcuate nucleus of the mouse

**DOI:** 10.1371/journal.pone.0213927

**Published:** 2019-03-27

**Authors:** Shel-Hwa Yeo, Victoria Kyle, Clemence Blouet, Susan Jones, William Henry Colledge

**Affiliations:** 1 Department of Physiology, Development and Neuroscience, University of Cambridge, Cambridge, United Kingdom; 2 MRC Metabolic Diseases Unit, University of Cambridge Metabolic Research Laboratories, WT-MRC Institute of Metabolic Science, University of Cambridge, Cambridge, United Kingdom; University of Cordoba, SPAIN

## Abstract

The normal function of the mammalian reproductive axis is strongly influenced by physiological, metabolic and environmental factors. Kisspeptin neuropeptides, encoded by the *Kiss1* gene, are potent regulators of the mammalian reproductive axis by stimulating gonadodropin releasing hormone secretion from the hypothalamus. To understand how the reproductive axis is modulated by higher order neuronal inputs we have mapped the afferent circuits into arcuate (ARC) *Kiss1* neurons. We used a transgenic mouse that expresses the CRE recombinase in *Kiss1* neurons for conditional viral tracing with genetically modified viruses. CRE-mediated activation of these viruses in *Kiss1* neurons allows the virus to move transynaptically to label neurons with primary or secondary afferent inputs into the *Kiss1* neurons. Several regions of the brain showed synaptic connectivity to arcuate *Kiss1* neurons including proopiomelanocortin neurons in the ARC itself, kisspeptin neurons in the anteroventral periventricular nucleus, vasopressin neurons in the supraoptic and suprachiasmatic nuclei, thyrotropin releasing neurons in the paraventricular nucleus and unidentified neurons in other regions including the subfornical organ, amygdala, interpeduncular nucleus, ventral premammilary nucleus, basal nucleus of stria terminalis and the visual, somatosensory and piriform regions of the cortex. These data provide an insight into how the activity of *Kiss1* neurons may be regulated by metabolic signals and provide a detailed neuroanatomical map for future functional studies.

## Introduction

Mammalian fertility requires pulsatile secretion of gonadotropin releasing hormone (GnRH) from the hypothalamus to stimulate the pituitary-gonadal axis [1]. Kisspeptin neuropeptides, produced by *Kiss1* neurons, are potent stimulators of GnRH secretion acting via the G-protein coupled receptor, KISS1R (also known as GPR54) expressed by GnRH neurons [2]. *Kiss1* neurons have been mapped to the arcuate nucleus (ARC) and the rostral periventricular area of the third ventricle (RP3V) of the hypothalamus, which includes the anteroventral periventricular (AVPV) nucleus [3, 4]. In rodents, *Kiss1* gene expression in the ARC is thought to regulate pulsatile GnRH release [5] while expression in the AVPV is responsible for the luteinizing hormone (LH) surge required for ovulation [6, 7]. Inactivating mutations in *Kiss1* or *Kiss1r* cause infertility in mice and humans [8–10].

Clearly *Kiss1* neurons play a critical role in regulating mammalian fertility but relatively little is known about the way in which these neurons are integrated within the neuronal circuitry of the hypothalamus or how the function of these neurons is co-ordinated with changes in physiological states. It is known that *Kiss1* neurons form functional connections with GnRH neurons but additional connections have also been identified to other regions of the hypothalamus and parts of the limbic system [11]. Defining the upstream inputs that regulate *Kiss1* activity has only just begun. Anterograde tracing has shown that vasopressin axons from the suprachiasmatic nucleus (SCN) form appositions with AVPV *Kiss1* neurons [12] although the significance of this is not clear since vasopressin does not increase the firing rate of *Kiss1* neurons [13]. Reciprocal connections between ARC *Kiss1* neurons and POMC and AgRP/NPY neurons have also been found [14, 15].

A fundamental step in understanding the neuroendocrine regulation of the reproductive axis requires accurate mapping of neuronal connectivity to *Kiss1* neurons. We have used a Kiss1-CRE transgenic mouse [16] to activate pseudorabies virus (PRV) transynaptic neuronal tracers specifically in ARC *Kiss1* neurons to define upstream connectivity with these neurons. We have identified a number of brain regions that form connections with ARC *Kiss1* neurons and are likely to be physiologically relevant including the amygdala, the paraventricular nucleus (PVN), the supraoptic nucleus (SON), the ventral tuberomammillary nucleus (VTM) and POMC neurons in the ARC.

## Materials and methods

### Animals

The Kiss1-CRE mice have been previously characterized [16]. Heterozygous mice aged between 2–4 months old were used for the viral tracing. *Kiss1–CRE; TdTomato* mice were obtained by crossing *Kiss1-CRE* and TdTomato reporter mice (*Gt(ROSA)26Sor–loxSTOPlox–TdTomato*; stock #007909; The Jackson Laboratory) [17, 18]. All mice were maintained on a 12:12-hours light-dark cycle (light on between 6:30 a.m. and 6:30 p.m.) with *ad libitum* access to food and water. Experimental procedures were performed under the authority of a United Kingdom Home Office Project License and approved by the Cambridge University Biomedical Services Local Ethics Committee.

### PRV construct and specificity

PRV Bartha 2001, Ba2001 (viral titre of 3.5 x 10^8^ pfu/ml) was kindly provided by Lynn Enquist (University of Princeton, USA). The viral construct is genetically engineered to contain a floxed-stop cassette between a CMV promoter and Tau-GFP-IRES-TK. The thymidine kinase (*tk*) gene is essential for viral replication. The cassette contains an SV40 polyadenylation signal and 5’ splice donor site that prevents expression of any downstream sequences unless it is removed by CRE-mediated recombination. When the virus infects CRE-expressing neurons, the stop signal is removed permanently, rendering the virus capable of replicating in other neurons in synaptic contact with the original neuron, and the infected neurons will express GFP. PRV Bartha Introvert; PRV^INTRO^ (viral titre 1.3 x 10^8^ pfu/ml) was kindly provided by Prof. Friedman (Rockefeller University, USA) prepared as previously described [19]. This viral construct consists of a lox-flanked transcriptional stop sequence, in which the tk gene was inverted between two inverted lox sites (Inverted-TK). Presence of CRE repairs the inversion, places a synthetic intron within the tk gene for activation, and enabling GFP expression in PRV-infected neurons. Viral specificity was tested by stereotaxic injection of both PRV vectors into the ARC of wild-type (n = 9) and Kiss1-CRE heterozygous animals (n = 10). The coordinates for injection were: anteroposterior, 1.60 mm, mediolateral, 0.2 mm and dorsoventral, 5.90 mm. Animals were sacrificed 24 or 32 hours (h) post-injection, brains were extracted after transcardial perfusion for post-hoc examination for PRV-GFP expression.

### Stereotaxic injections

Mice were anaesthetized with 2.0–3.5% isoflurane and placed in a stereotaxic apparatus (David Kopf Instruments, Tujunga, CA, USA). A scalpel was used to open an incision along the midline to expose the skull. After performing a craniotomy, either 0.75 μL of PRV or 0.4 μl of AAV5-hSyn-hChR2-eYFP at a titre of 4.8 × 10^12^ particles per ml was bilaterally injected per site (ARC or PVN) using a 1.0 μl Neurosyringe (Hamilton Syringes, Nevada, USA) at 0.1 μl min^−1^. The syringe was coupled to a 32 gauge flat-end needle. The syringe was slowly retracted 20 min after the start of the infusion. A slow injection rate followed by 10 min of waiting before retracting the syringe is crucial to restrict viral expression to the ARC or PVN. Mid-caudal arcuate coordinates were anteroposterior, −2.0 mm, mediolateral, +0.6 mm, dorsoventral, −5.5 mm. The coordinates for the PVN injections were: anteroposterior, -0.60 mm; mediolateral, +0.25 mm; dorsoventral, -4.60 mm. For colchicine treatment, animals were given PRV injections and allowed to recover for 5 days prior to colchicine administration (0.8 μg per g body weight) into the lateral ventricle (anteroposterior, -0.1 mm; mediolateral, +1.20 mm; dorsoventral, -2.25 mm. All coordinates were measured from the bregma.

### Monosynaptic tracing with a rabies vector

The AAV8-hSyn-FLEX-TVA-P2A-eGFP-2A-oG and EnvA G-deleted Rabies-mCherry were acquired from the UNC Vector Core (Salk Institute virus core, US). Kiss1‐Cre male mice (n  =  4) and wild type controls (n  =  2) underwent stereotaxic injection as mentioned previously. Using a Hamilton syringe, 0.6 μl of AAV8-hSyn-FLEX-TVA-P2A-eGFP (3.64 × 10^13^ viral genomes/ml) was injected unilaterally into the mid-caudal arcuate. After the injection, the syringe was left in the injection site for 10 min to minimize back flow of the vector into the needle tract. Animals recovered after the first injection. Three weeks later, the same stereotaxic procedure was repeated with 0.6 μl of the EnvA G-deleted Rabies (3.78 × 10^7^ transforming units/ml). Five days after the rabies vector injection, the mice were deeply anesthetized with an overdose of sodium pentobarbital and transcardially perfused with 4% paraformaldehyde.

### Tissue preparation

Mice were allowed to recover for a period of 32 h to 6 days after viral delivery, anaesthetized with an overdose of pentobarbital (3 mg/ kg body weight per 100 μL) and perfused transcardially with 15 mL of 4% PFA in 0.1 M phosphate buffered saline (Sigma-Aldrich, UK) at pH 7.6. The brains were removed from the skull, post-fixed in the same fixative at room temperature for 1 hour and then transferred to 30% sucrose/Tris-buffered saline (TBS; 25 mm Tris, 0.85% NaCl, pH 7.6; Sigma-Aldrich, UK) for cryoprotection. Three sets of 50 μm thick coronal brain sections were cut from the level of the medial septum through to the hindbrain for free-floating immunohistochemistry.

### Immunohistochemistry

The primary antibodies used in immunohistochemical studies are listed in Table 1. Chromagen labelling was applied to detect GFP or mCherry-expressed neurons after PRV and Rabies infection. Sections were treated with 3% hydrogen peroxide for 10 min to quench endogenous peroxidase activity. The sections were washed for 10 min for 3 times in TBS and then incubated for 48 h at 4°C with primary antibodies (refer to Table 1) added to the incubation solution containing TBS, Triton-X-100, bovine serum albumin and 2% normal serum from the species in which the secondary antibody was raised. All subsequent incubations were performed with the same incubation solution. After washing 3 times (10 min each) in TBS, the sections were incubated with biotinylated secondary immunoglobulins (IgG; Vector Labs, US) directed against the species in which the primary antibodies were raised for 90 min at room temperature. After three subsequent washes, sections were incubated with Vector Elite avidin-peroxidase (1:100, Vector Labs, US) for 90 min at room temperature. Finally, the sections were rinsed in TBS and immunoreactivity was revealed using glucose oxidase and nickel-enhanced diaminobenzidine hydrochloride (NiDAB; Sigma-Aldrich, UK) that generates a black precipitate in the nucleus or cytoplasm of the labelled cells. The sections were rinsed thoroughly and were immediately mounted, air-dried, dehydrated in ethanol followed by xylene (Sigma-Aldrich, UK) and a cover slip applied with DPX (BDH, UK).

**Table 1:** Antibodies used for immunostaining.

Antibody	Species	Source	Catalogue No.	Dilution	RRID
GFP	Chicken	Thermo Fisher Scientific	PA1-86341	1:1000 (NiDAB) 1:2000 (IF)	AB_931091
mCherry	Mouse	Abcam	Ab125096	1:2000	AB_11133266
Kisspeptin-10	Rabbit	Alain Caraty, INRA, France	AC566	1:2000	AB_2314709
POMC	Rabbit	Phoenix Pharmaceuticals	H-029-30	1:4000	AB_2307442
AVP	Rabbit	Millipore	AB1565	1:2000	AB_90782
TRH	Rabbit	Progen Biotechnik	11170	1:2000	AB_1543069
CRH	Rabbit	Immunostar	20084	1:2000	AB_572228
Oxytocin	Rabbit	Immunostar	20068	1:2000	AB_572258
c-Fos	Rabbit	Santa Cruz Biotechnology	SC-166940	1:500	AB_10609634

Free-floating, immunofluorescence labelling was performed to visualize the co-labelling of GFP-expressing neurons and neuropeptides on the coronal brain sections of animals received PRV injections. Sections were incubated overnight with the primary antibodies (Table 1) added with 2% normal donkey serum in TBS containing 0.3% Triton-X-100 and 0.25% Bovine Serum Albumin (BSA). After several washes with TBS, the sections were placed in biotinylated secondary immunoglobulins (1:200; Jackson ImmunoResearch) against the species in which the primary antibodies were raised and then incubated with combination of AlexaFluor-488-conjugated streptavidin and Alexa Fluor-568-conjugated immunoglobulins, each for 90 min at room temperature (RT) (1:200; Molecular Probe, UK). All sections were then washed, mounted on slides, air dried, and cover slipped with Vectashield Fluorescence Mounting Medium (Vector Labs, US). Negative controls by means of omission of primary and/or secondary antibodies for the different combinations, and these sections did not exhibit the appropriate immunofluorescence.

Staining against c-FOS was performed similarly to the procedure described above, but with the alterations described below. Brain slices were washed 3 X 30 min in 1X TBS + 1% Triton-X. The slices were treated for antigen retrieval with Citrate Buffer (pH 6; Sigma-Aldrich; UK) at 80°C for 45 min. The first incubation was using anti-c-FOS sc-52 made in Rabbit (catalogue number sc-166940; Santa Cruz, US) at 1:500 in 0.6% Triton-X in 3% normal goat serum, and it lasted 72 h at 4°C. The secondary antibody was goat anti-rabbit immunoglobulin conjugated with DyLight 405 (Thermo Fisher, cat. no. 150075, UK), incubated at a 1:500 dilution for 12 h at 4°C. The slices were washed, mounted on slides, air dried, and cover slipped with Vectashield Fluorescence Mounting Medium.

### Image analysis

All fluorescent images were generated using a Leica TCS SP2 Laser Scanning Confocal Microscope (Cambridge Advanced Imaging Centre, Cambridge, UK). Images were captured using a x40/1.1 or a x63/1.2 water immersion objectives. Dylight 405, Alexa Fluor 488 and TdTomato (568) were excited with 405, 488, and 561 nm laser lines and emission collected with 395 to 415 nm, 500 to 550 nm and 580 to 620 nm bandpass emission filters, respectively. All images were captured using sequential scanning mode and image stacks were collected with 1.0 μm focus intervals. Eight bit confocal images were acquired with a 512 x 512 pixels format, a scan speed of 400 Hz. All images were digitally processed with Adobe Photoshop (Adobe System), where the levels of brightness and contrast were adjusted to enhance the quality of images. As for chromagen labelled sections, images were acquired using a Zeiss Axio Imager A1 microscope (Centre for Trophoblast Research, Cambridge, UK). The number of PRV-GFP-stained or other antibody-stained neurons/cell bodies was counted in each region of two brain sections for each animal and indicated as mean number with standard error of mean (SEM).

### Optogenetic stimulation

Four to five weeks after the animals (n = 3 males) were injected with AAV5-hSyn-hChR2-eYFP (University of North Carolina vector core, US) they were culled by cervical dislocation and brain slices of 250 μm cut using a Campden 7000smz Vibrating Microtome. Slices were supplied with 90% oxygen and 5% carbon dioxide in artificial cerebrospinal fluid (ACSF) for 1 hour prior to experiment. Slices were transferred to the stage of an Olympus BX51W upright microscope and PVN-GFP neurons or Kiss1-Tdtomato neurons were viewed using the relevant filters. The chamber was perfused at 2–3 ml min^-1^ with ACSF. For c-FOS experiments, trains of blue light pulses at 20 Hz for 20s epoch and 10s between sweeps for 20 min were applied to ARC slices, and were allowed to recover for 1 h in the chamber. Control slices were incubated in the chamber without light stimulation. Slices were fixed with 4% PFA for three hours at RT prior to immunostaining. For electrophysiological recordings, patch pipettes were filled with a Cs-based intracellular solution, cells were voltage clamped using an Axopatch 200B amplifier and membrane current was low pass filtered at 2 kHz and sampled at 20 kHz using a Micro 1401 controlled by Spike 2 (Version 4) software (Cambridge Electronic Design). Pulses of blue light (480 nm, 10ms) were applied to activate ChR2.

## Results

### Validation of PRV-GFP specificity and activation restricted to *Kiss1* neurons

Specific CRE-activation of pseudorabies viruses (PRV) has been widely used to precisely map CNS circuits from molecularly defined neurons [20]. We used both PRV Ba2001 and PRV^INTRO^ [19] in these studies but the PRV^INTRO^ gave more robust CRE-dependent GFP expression compared to PRV Ba2001. Therefore, the Ba2001 virus was mainly used to confirm the specificity of viral activation and to map the local circuits in the arcuate region of the hypothalamus. Most tracing data were obtained from PRV^INTRO^ experiments unless otherwise stated.

**Fig 1:**
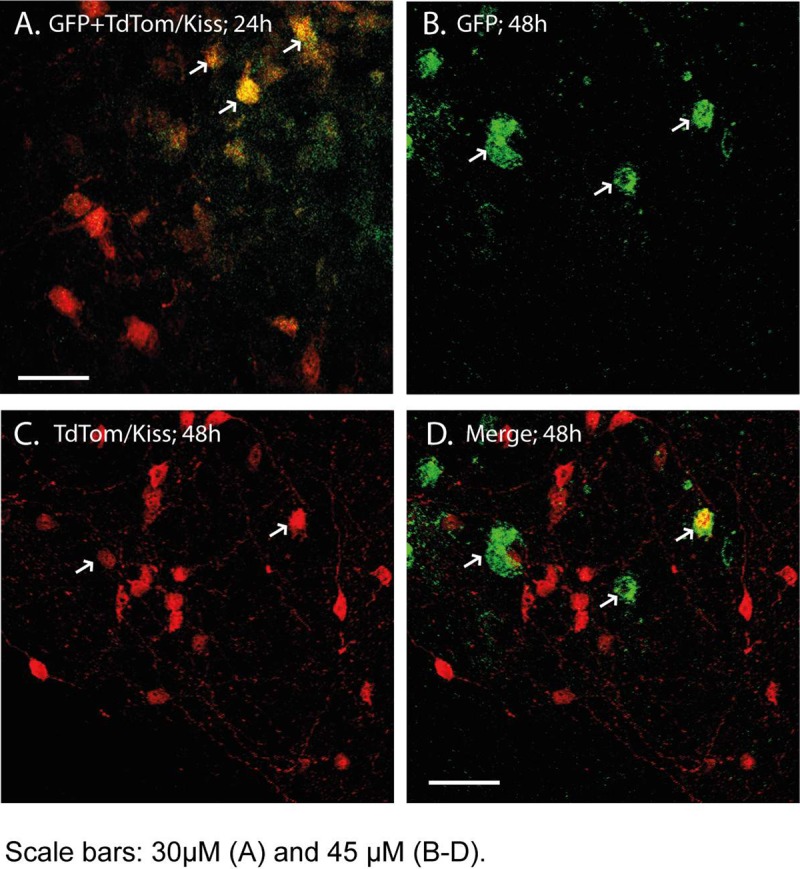
CRE-dependent PRV-GFP activation in *Kiss1* neurons. Stereotaxic delivery of the PRV^INTRO^ virus into the arcuate (ARC) region of mice was associated with GFP expression only in Kiss1-CRE mice. PRV^INTRO^ driven GFP expression (arrowed) at 24 h after delivery was restricted to *Kiss1* neurons (A) but at 48 h expression was also found in non-*Kiss1* neurons (B-D). Scale bars 30 μm (A) and 45 μm (B-D).

24 h after PRV^INTRO^ delivery, GFP expression was restricted to *Kiss1* neurons (Fig 1A) with 100% of GFP positive neurons co-labelled with TdTomato (5 ± 2; n = 2; Table 2), which is a marker of *Kiss1* neurons. At later time points, GFP expression was found in non-*Kiss1* neurons (Fig 1B–1D) with only 53% of GFP positive neurons showing TdTomato expression at 48 h (9 ± 3; n = 4; Table 2). PRV injections into the ARC of wild-type mice did not give GFP-expressing cells for PRV Ba2001 (S1 Fig) or PRV^INTRO^ (data not shown).

**Table 2:** Time course of spread of PRV^INTRO^-GFP from *Kiss1* neurons into adjacent neurons in the arcuate.

Time Course	24 hours (n = 2)	32 hours (n = 4)	48 hours (n = 4)
No. of GFP positive neurons	5 ± 2	12 ± 5	19 ± 6
No. of GFP positive neurons expressing TdTomato/kisspeptin	5 ± 2	8 ± 3	10 ± 3
No. of GFP positive neurons expressing POMC	NA	4 ± 2	9 ± 3

### Brain areas with inputs into ARC *Kiss1* neurons

**Fig 2:**
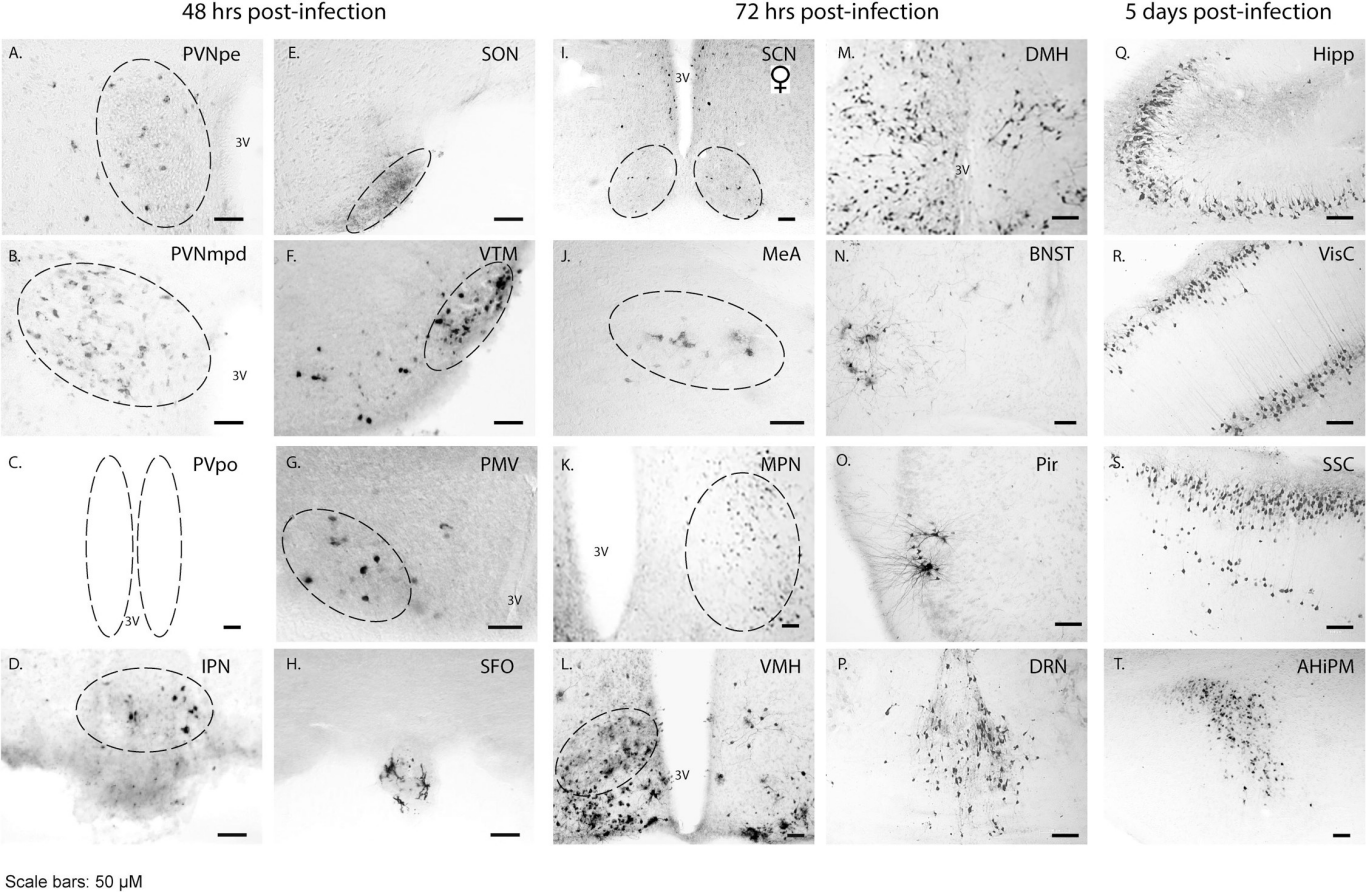
Time course of PRV spread. Following activation of the PRV^INTRO^ vector in arcuate *Kiss1* neurons, the spread of the virus into upstream neurons was visualized at different times by immunohistochemistry for GFP expression. AHiPM, amygdalohippocampal area; BNST, bed nucleus of the stria terminalis; DMH, dorsomedial hypothalamus; DRN, dorsal raphe nucleus; Hipp, hippocampus; IPN, interpeduncular nucleus; MeA, medial amygdala; MPN, medial preoptic nucleus; Pir, piriform cortex; PMV, ventral premammillary nucleus; PVNmpd, paraventricular nucleus medial posterodorsal; PVNpe, paraventricular nucleus periventricular part; PVpo, periventricular preoptic nucleus; SCN, suprachiasmatic nucleus (female only); SFO, subfornical organ; SON, supraoptic nucleus; SSC, somatosensory cortex; VisC, visual cortex; VMH, ventral medial hypothalamus; VTM, ventral tuberomammillary nucleus; 3V, 3^rd^ ventricle. Scale bars 50 μm.

The distribution of PRV^INTRO^-GFP positive neurons was mapped in brain slices at different times after PRV delivery into the ARC. The temporal spread of the PRV^INTRO^-GFP virus gives an approximate indication of whether the neuronal inputs into the *Kiss1* neurons are primary or secondary as it takes around 24–48 h for the PRV to transverse each synaptic connection (Fig 1B–1D). 48 h after viral delivery, PRV-GFP expression was found in the arcuate, the paraventricular nucleus (both the medial posterodorsal and periventricular regions), the ventral premammillary nucleus, the supraoptic nucleus, the ventral tuberomammillary nucleus, the periventricular preoptic nucleus in the hypothalamus, the interpeduncular nucleus in the midbrain and the circumventricular subfornical organ (Fig 2A–2H), suggesting regions with primary afferent inputs to *Kiss1* neurons. 72 h after viral delivery, indicating regions with possibly secondary or tertiary afferent inputs, PRV-GFP expression was found in the suprachiasmatic nucleus, the dorsomedial hypothalamus, the medial amygdala, the bed nucleus of the stria terminalis, the medial preoptic nucleus, the piriform cortex, the ventral medial hypothalamus and the dorsal raphe nucleus (Fig 2I–2P). At 5 days post-infection, PRV-GFP spread was also found at sites further away from the hypothalamus such as the hippocampus, the visual and somatosensory cortex regions and the amygdalohippocampal region (Fig 2Q–2T). A similar distribution of PRV-GFP virus was observed using both PRV Ba2001 and PRV^INTRO^ apart from the supraoptic nucleus (SON) and the medial amygdala regions, which were only found using the PRV^INTRO^ probably due to its higher titre. No obvious difference between males and females was found in the number of PRV-GFP positive neurons in most regions except for the suprachiasmatic nucleus (Table 3).

**Table 3:** Quantification of PRV^INTRO^-GFP positive neurons in different regions across different time points.

No. of PRV-GFP positive cells in different brain regions	Males (n = 5–9)	Female (n = 6–9)
**48 hours**		
ARC	49 ± 12	68 ±8
PVN (mpd)	70 ± 20	77 ± 15
PVN (pe)	66 ± 11	58 ± 9
SON	30 ± 10	26 ± 7
MPN	28 ± 13	30 ± 17
PVpo	32 ± 10	40 ± 9
VTM	19 ± 6	17 ± 4
**72 hours**		
PMV	28 ± 7	30 ± 9
SCN	0	11 ± 5
MeA	8 ± 3	5 ± 2
PAG	13 ± 4	10 ± 3
DRN	19 ± 8	21 ± 7
IPN	8 ± 3	6 ± 3
SFO	4 ± 2	3 ± 1

DRN, dorsal raphe nucleus; IPN, interpeduncular nucleus; MeA, medial amygdala; MPA, medial preoptic area, MPN, medial preoptic nucleus; PAG, periaquaductal grey; PVNmpd, paraventricular nucleus medial posterodorsal; PVNpe, paraventricular nucleus periventricular part; PMV, ventral premammillary nucleus; PVpo, periventricular preoptic nucleus; SCN, suprachiasmatic nucleus; SFO, subfornical organ, SON, supraoptic nucleus; VTM, ventral tuberomammillary nucleus.

### Neuropeptide identity of neurons with synaptic connections to ARC *Kiss1* neurons

**Fig 3:**
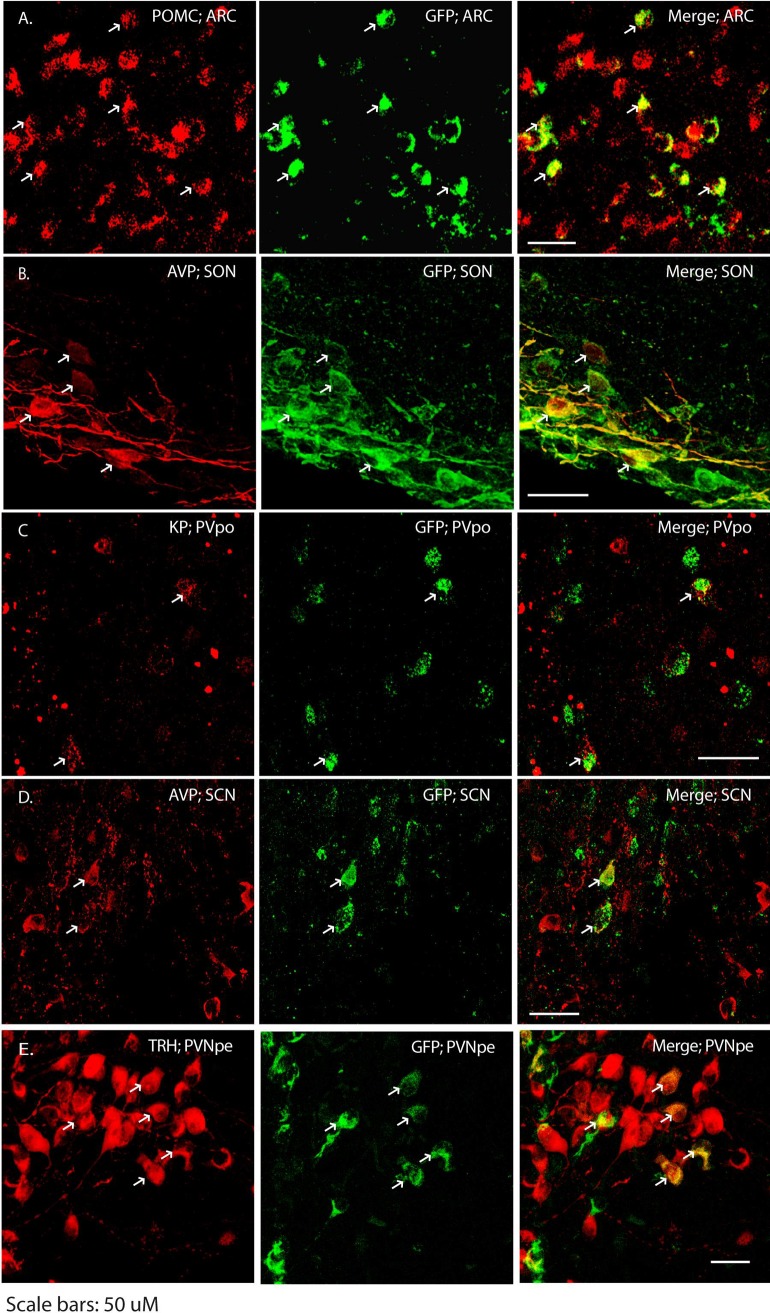
Identity of neurons connecting to ARC *Kiss1* neurons. Following PRV^INTRO^ (A-B) and PRV Ba2001 (C-E) spread from ARC *Kiss1* neurons, immunohistochemistry was performed on brain sections to identify neuropeptides (red) that co-localized with GFP (green). POMC, pro-opiomelanocortin; KP, kisspeptin; AVP, arginine vasopressin; TRH, thyrotropin releasing hormone. ARC, arcuate nucleus; PVpo, periventricular preoptic nucleus; SCN, suprachiasmatic nucleus; SON, supraoptic nucleus; PVNpe, paraventricular nucleus periventricular part. Scale bars 50 μm.

To determine the identity of primary/secondary neuronal projection to *Kiss1* neurons, we used immunohistochemistry to examine co-localization between PRV-GFP and neuropeptides that are expressed by neurons in the regions to which the PRV spread. All co-labelling experiments were performed using brain sections derived from PRV Ba2001-injected animals apart from the pro-opiomelanocortin and vasopressin immunostaining in the ARC and SON, respectively. These were done in the PRV^INTRO^-infected animals. Within the arcuate, we found that the PRV-GFP spread into pro-opiomelanocortin neurons (Fig 3A) while in the periventricular preoptic region, several PRV-GFP positive neurons expressed kisspeptin (Fig 3C). Arginine vasopressin neurons (AVP) were found to co-localize with PRV-GFP in the supraoptic (Fig 3B) and the suprachiasmatic nucei (Fig 3D). Several neuronal types are found in the paraventricular nucleus (PVN). We found 56 ± 17% (n = 3 males, 72 h post infection) of PRV-GFP positive neurons expressed thyrotropin releasing hormone, TRH (Fig 3E). In contrast, we did not observe any GFP co-localization in vasopressin, oxytocin, or corticotropin releasing hormone (CRH) neurons in the PVN (**S2 Fig**). The proportions of PRV-GFP neurons showing co-localization with the different neuropeptides is given in Table 4.

**Table 4:** Quantification of PRV Ba2001-GFP positive neurons showing co-localization with neuropeptide markers.

Brain Region/neuropeptide (No. of mice analysed)	No. of GFP positive neurons	No. of GFP positive neurons expressing neuropeptide (%)
ARC/POMC (2 Females; 2 Males)*	19 ± 6	9 ± 3 (47% ± 12)
PVpo/Kisspeptin (3 Females)	20 ± 11	8 ± 3 (40% ± 15)
SCN/AVP (3 Females)	12 ± 7	4 ± 2 (33% ± 17)
SON/AVP (2 Males; 2 Females)*	40 ± 12	32 ± 6 (80% ± 15)
PVN/TRH (3 Males)	48 ± 22	27 ± 8 (56% ± 17)

* **PRV**^**INTRO**^**-GFP**

### Non-specific optogenetic activation of PVN neurons did not stimulate ARC *Kiss1* neurons

**Fig 4:**
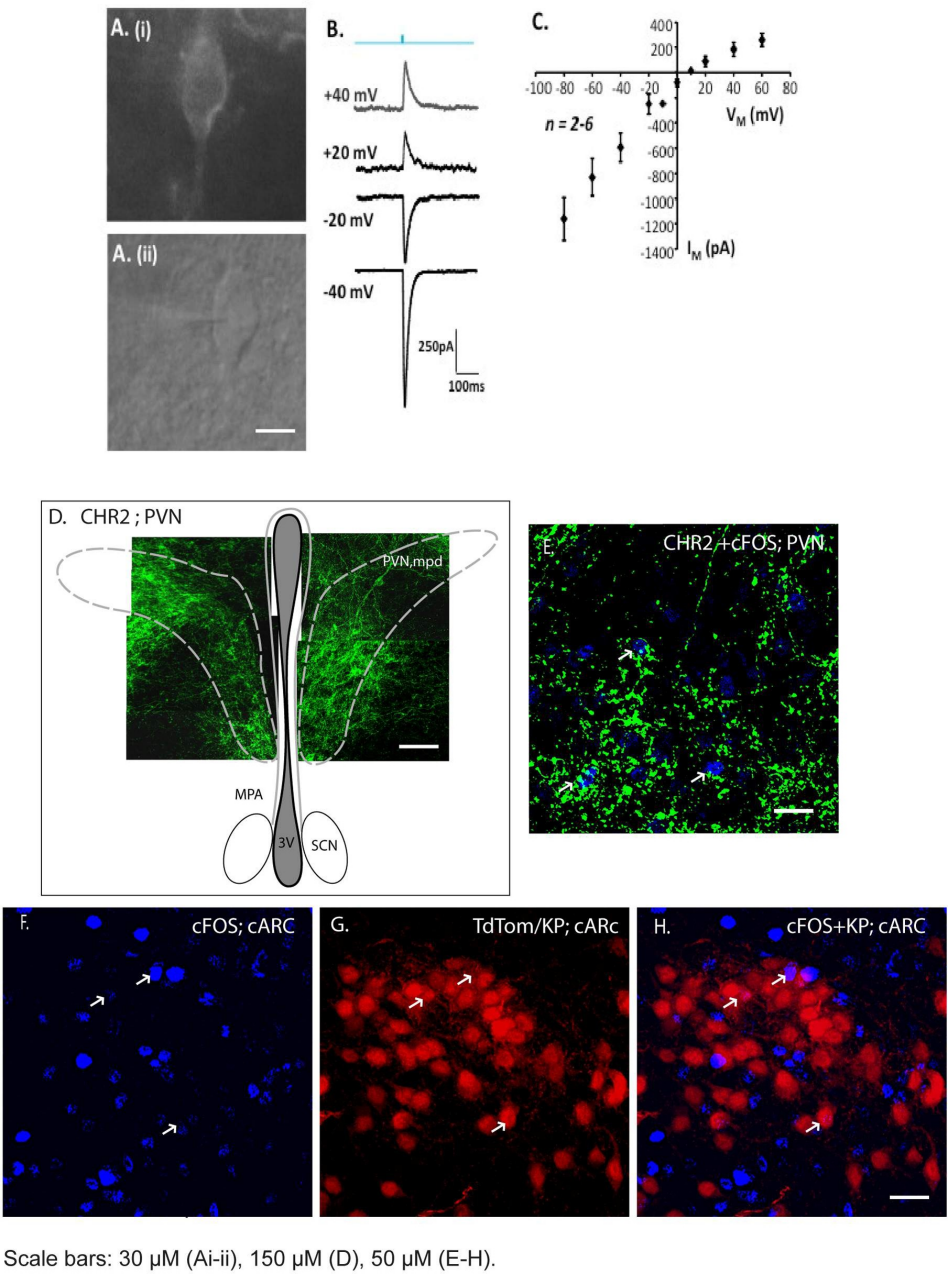
AAV-ChR2 delivery to PVN neurons and optogenetic responses in the ARC. AAV-ChR2 was delivered by stereotaxic injection into the PVN and 8 weeks later, brain slices were stimulated by blue light to evaluate direct synaptic connectivity between PVN neurons and *Kiss1* neurons in the arcuate. A. Expression of AAV-ChR2-GFP in PVN neurons 8 weeks after stereotaxic injection into PVN (i) GFP (ii) Light. B. Example responses of a single PVN neuron to brief light stimulation (blue; 480 nm, 10 ms duration) at different membrane potentials. C. Current voltage relationship for light activated responses in PVN neurons. D. A composite of confocal images indicating neurons expressing ChR2 within the medial posterodorsal portion of the PVN. E. Robust expression of the AAV-ChR2 vector was confirmed by GFP immunofluorescence and c-FOS induction (blue, arrowed) in the PVN after light stimulation (Fig 4E). F. c-FOS expression in the caudal ARC after light stimulation. G. *Kiss1* neurons in the caudal ARC visualized by TdTomato expression. H. Merged image showing limited activation of c-FOS expression in *Kiss1* neurons. ARC, arcuate nucleus; PVN, paraventricular nucleus; ac, anterior commissure. MPA, medial preoptic are; PVNmpd, paraventricular nucleus medial posterodorsal; SCN, suprachiasmatic nucleus; 3V, 3^rd^ ventricle. Scale bars: 30 μM (Ai-ii), 100 μM (D), 50 μM (E-H).

Since we found a large number of neurons in the PVN that were labelled by the activated PRV^INTRO^-GFP vector, we wanted to establish whether these neurons formed functional connections with ARC *Kiss1* neurons. To do this, we injected the AAV5-hSyn-hChR2-eGFP vector into the PVN, targeting the periventricular and medial posterodorsal parts and looked for electrophysiological activation of *Kiss1* neurons in brain slices after optogenetic stimulation. We evaluated several AAV-ChR2 serotypes to identify one that gave the most extensive expression in the PVN. The AAV5-hSyn-hChR2-eGFP resulted in bilateral GFP expression in 69 ± 4 neurons within the PVN (2 sections/animal; n = 2). Expression of the AAV-ChR2 vector was confirmed by extensive GFP fluorescence in the PVN of the brain slices (Fig 4D). Blue light was used to activate ChR2-expressed in soma within the PVN or nerve terminals in the ARC. Membrane current was recorded from GFP positive neurons in the PVN and TdTomato positive *Kiss1* neurons in the ARC at different membrane potentials. Although light-evoked responses were found in PVN neurons and showed the expected current-voltage relationship for the non-selective cation conductance mediated by ChR2 (Fig 4B and 4C), no light-evoked responses were detected in *Kiss1* neurons (14 neurons from 4 mice). To address whether this was due to a lack or a low level of connectivity between PVN and *Kiss1* neurons, we also measured activation of c-FOS after light stimulation (Fig 4F–4H) to measure simultaneous activation of a greater number of *Kiss1* neurons. There was a ten-fold increase in the number of c-FOS expressing neurons in the arcuate after light stimulation indicating good connectivity from the PVN, but only around 5% of *Kiss1* neurons expressed c-FOS (Table 5). These data suggest that PVN neurons connect to neurons within the arcuate, upstream of *Kiss1* neurons, rather than directly to *Kiss1* neurons themselves.

**Table 5:** Optogenetic activation of c-FOS in the ARC after AAV-ChR2 delivery to the PVN (n = 3 males).

Brain Slice Region	Photostimulation	No. of TdTomato neurons	No. of c-FOS neurons	No. of TdTomato + c-FOS neurons
rostral ARC	Yes	91 ± 53	162 ± 61	3 ± 2
medial ARC	Yes	106 ± 25	142 ± 36	0
caudal ARC	Yes	197 ± 65	169 ± 60	9 ± 5
Negative Control				
medial ARC	No	88 ± 37	16 ± 7	0

### Distinguishing primary from secondary afferents into ARC *Kiss1* neurons

**Fig 5:**
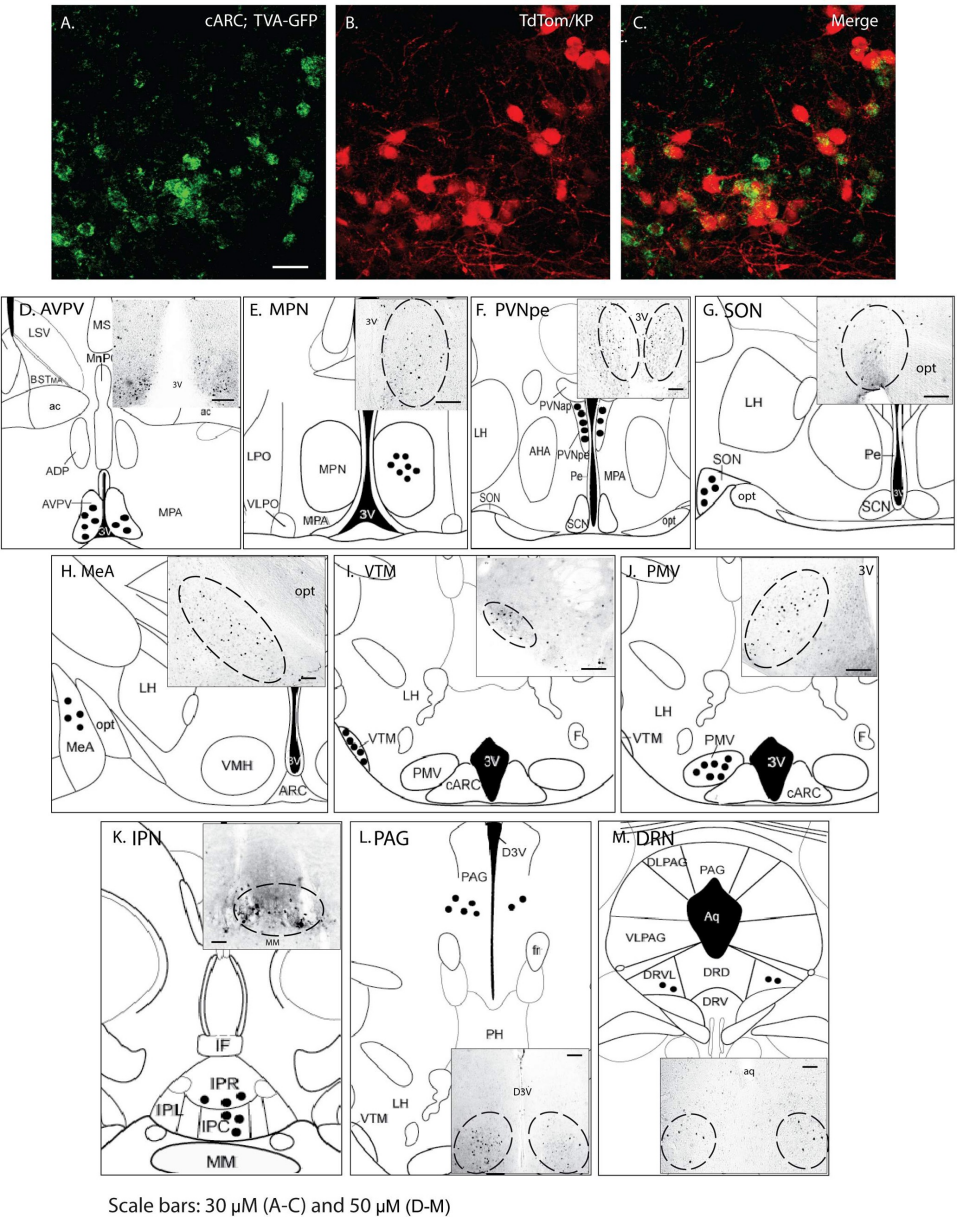
Distinguishing primary from secondary afferents into ARC *Kiss1* neurons. (A) AAV8-hSyn-FLEX-TVA-P2A-eGFP expression was confirmed in caudal ARC *Kiss1* neurons prior to rabies viral vector injection. (D-M) Schematic diagrams and images indicating brain regions with mCherry expression (black circles) indicating monosynaptic inputs to caudal ARC *Kiss1* neurons. ADP, anterodorsal preoptic nucleus; AHA, anterior hypothalamic area; ARC, arcuate nucleus; AVPV, anteroventral periventricular nucleus; BSTMA, bed nucleus of the stria terminalis, medial division; CARC, caudal arcuate nucleus; D3V, dorsal 3rd ventricle; DLPAG, dorsolateral periaqueductal gray; DRD, dorsal raphe nucleus, dorsal part; DRN, dorsal raphe nucleus; DRV, dorsal raphe nucleus, ventral part; DRVL, dorsal raphe nucleus, ventrolateral part; F, fornix; IF, interfascicular nucleus; IPC, interpeduncular nucleus, caudal subnucleus; IPL, interpeduncular nucleus, lateral subnucleus; IPN, interpeduncular nucleus; IPR, interpeduncular nucleus, rostral subnucleus; LH, lateral hypothalamic area; LPO, lateral preoptic area; LSV, lateral septal nucleus, ventral part; MeA, medial amygdala; MPA, medial preoptic area; MPN, medial preoptic nucleus; MM, medial mammillary nucleus; PAG, periaquaductal grey; Pe, periventricular nucleus; PH, posterior hypothalamic area; PVNap, paraventricular nucleus, anterior parvicellular part; PVNpe, paraventricular nucleus periventricular part; PMV, ventral premammillary nucleus; SCN, suprachiasmatic nucleus; SON, supraoptic nucleus; VMH, ventromedial hypothalamic nucleus; VLPAG, ventrolateral periaqueductal gray; VLPO, ventrolateral preoptic nucleus; VTM, ventral tuberomammillary nucleus; 3V, 3^rd^ ventricle; aq, aquaduct; ac, anterior commissure; fr, fasciculus retroflexus; opt, optic tract. Scale bars: 30 μm (A-C) and 50 μm (D-M).

Although the temporal spread of the activated PRV-GFP virus provides some indication about whether the inputs to *Kiss1* neurons are primary or secondary, the data are not unequivocal. For example, as illustrated above, secondary inputs can be mistaken as primary if the virus rapidly spreads via another neuron in close proximity to the *Kiss1* neurons. In addition, one reason for the lack of optogenetic activation of *Kiss1* neurons after ChR2 delivery to the PVN might be because these connections are from higher order inputs. To establish which of the regions represent primary afferent inputs, we used a CRE-dependent rabies virus as a monosynaptic tracer [21]. This method uses a combination of a CRE-dependent helper virus and an EnvA-pseudotyped, G-deleted rabies virus to identify direct inputs to ARC *Kiss1* neurons. Fifty one percent of the mid-caudal arcuate kisspeptin neurons expressed the TVA-GFP/ TVA-receptor protein as the precursor neurons for rabies infection whereas 75% of the TVA-GFP expressing cells in the mid-caudal ARC were *Kiss1* neurons. No GFP fluorescence was observed from the AAV8-hSyn-FLEX-TVA-P2A-eGFP-2A-oG vector in wild-type mice indicating that viral activation is CRE dependent and we are probably not visualizing all the *Kiss1* neurons that show viral activation. We found direct inputs from neurons in the anteroventral periventricular nucleus; the dorsal raphe nucleus; the interpeduncular nucleus; the medial amygdala (MeA); the medial preoptic area (MPA); the periaquaductal grey; the periventricular part of the paraventricular nucleus; the ventral premammillary nucleus the supraoptic nucleus; dorsal raphe nucleus, and the ventral tuberomammillary nucleus (Fig 5). No primary afferent inputs from the suprachiasmatic nucleus were found. The number of Rabies-mCherry positive neurons in different regions is listed in Table 6.

**Table 6:** Quantification of rabies-mCherry positive neurons in different regions.

No. of Rabies-mCherry positive neurons in different regions	Males (n = 3)
AVPV/PVpo	37 ± 11
MPA	29 ± 10
MPN	34 ± 14
PVN (pe)	68 ± 19
SON	11 ± 2
MeA	17± 8
VTM	14 ± 4
PMV	42 ± 18
IPN	15 ± 6
PAG	28 ± 9
DRN	14 ± 4

AVPV, anteroventral periventricular nucleus; DRN, dorsal raphe nucleus; IPN, interpeduncular nucleus; MeA, medial amygdala; MPA, medial preoptic area, MPN, medial preoptic nucleus; PAG, periaquaductal grey; PVNpe, paraventricular nucleus periventricular part; PMV, ventral premammillary nucleus; PVpo, periventricular preoptic nucleus; SCN, suprachiasmatic nucleus; SFO, subfornical organ, SON, supraoptic nucleus; VTM, ventral tuberomammillary nucleus.

## Discussion

This is the first study showing the extensive connections of primary and secondary inputs to *Kiss1* neurons in the arcuate region of the hypothalamus. *Kiss1* neurons are critical for mammalian fertility and in the arcuate (ARC) they have a vital role in the generation of pulsatile GnRH release, which is required to maintain the reproductive axis. Since fertility is modulated by many physiological and environmental factors, these studies were aimed at determining the neuronal inputs to arcuate *Kiss1* neurons to identify novel pathways that might regulate the reproductive axis.

To determine the hierarchical control of *Kiss1* neuronal regulation, we mapped neural inputs from other CNS centres onto *Kiss1* neurons in the ARC region of the hypothalamus using conditionally attenuated pseudorabies viruses (PRV) as well-established neural tracers [22]. A particular strength of this approach compared to other neuronal tracing methods is that the virus moves across synaptic connections so that the circuits that are identified are likely to be functionally relevant.

**Fig 6:**
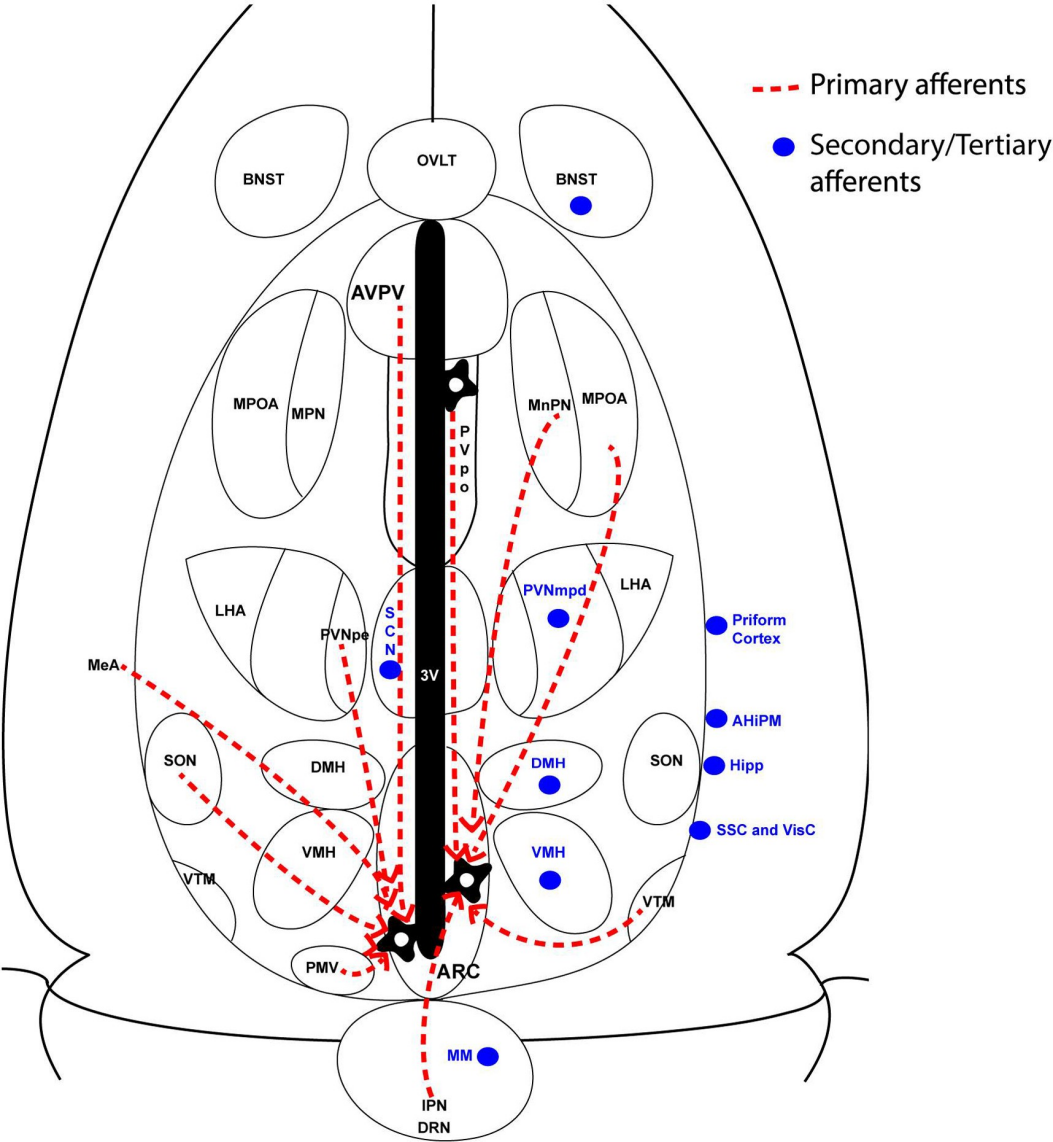
Summary of neuronal inputs into ARC *Kiss1* neurons. Red dashed lines represent primary afferents whereas blue circles represent secondary afferents to mid-caudal ARC *Kiss1* neurons. AHiPM, amygdalohippocampal area; BNST, bed nucleus of stria terminalis; DMH, dorsomedial hypothalamus; DRN, dorsal raphe nucleus; Hipp, hippocampus; IPN, interpeduncular nucleus; MeA, medial amygdala; MM, mammillary nucleus; MPOA, medial preoptic area; MPN, median preoptic nucleus; PMV, ventral premamillary nucleus; PVNmpd, paraventricular nucleus (medial posterodorsal part); PVNpe, paraventricular nucleus (periventricular part); SCN, suprachiasmatic nucleus; SON, supraoptic nucleus; VMH, ventromedial hypothalamus; VTM, ventral tuberomammillary nucleus;

After stereotaxic delivery of the PRV-GFP vectors (mainly using PRV^INTRO^) into the arcuate of Kiss-CRE mice, we mapped regions of the brain with synaptic connections to ARC *Kiss1* neurons. Since PRV moves across synapses exclusively in a retrograde manner, we were able to identify a subset of neurons and circuits located upstream of the *Kiss1* neurons in the arcuate. Monosynaptic tracing was then used to distinguish primary from secondary inputs. We identified several regions with inputs into the *Kiss1* neurons and these are summarized in Fig 6. Many of these regions have not been shown to form connections with *Kiss1* neurons previously and therefore represent novel inputs that may regulate the activity of *Kiss1* neurons.

PRV vectors take around 24–48 h to move across each synaptic connection in a neuronal circuit [19], so the temporal spread of the virus provides an approximate indication of whether the inputs to the *Kiss1* neurons are primary, secondary or higher. In principle, 72 h to five days post-infection should represent secondary or tertiary connections. At 24 h after viral delivery, PRV^INTRO^ expression was limited to *Kiss1* neurons in the ARC but by 48 h had moved to non-*Kiss1* neurons, likely representing primary inputs into the *Kiss1* neurons. We hypothesised that some of these neurons may communicate information about metabolic status to *Kiss1* neurons using leptin or ghrelin as a signal. Under conditions of negative energy balance, *Kiss1* expression in the ARC is reduced in pre-pubertal rats [23] but *Kiss1* neurons do not respond directly to leptin [24]. During starvation there are also changes in expression of the anorexigenic peptide α-melanocyte-stimulating hormone (α-MSH) from POMC neurons and the orexigenic peptides neuropeptide Y (NPY) and agouti-related peptide (AGRP) from NPY/AgRP neurons [25]. We hypothesised that NPY and POMC neurons connect to ARC *Kiss1* neurons to regulate kisspeptin expression under conditions of negative energy balance. Indeed, we found that a subset of POMC-expressing neurons in the ARC was labelled with PRV-GFP indicating that they form primary afferents to *Kiss1* neurons. Glutamatergic input from ARC *Kiss1* neurons to POMC neurons [14] as well as the role of POMC neurons in mediating leptin-α-MSH-kisspeptin signalling pathway in pre-pubertal female rodents [26] have been reported and our data are consistent with these studies.

Padilla *et al*. have shown that ARC *Kiss1* neurons receive less inhibitory input after ablation of AgRP/NPY neurons in mice and that optogenetic stimulation of AgRP neurons can induce inhibitory post-synaptic currents (IPSC) in *Kiss1* neurons [15]. These data indicate that there are direct, GABAergic connections from AgRP neurons to some *Kiss1* neurons. Fluorescence *in situ* hybridization will be required to validate this in the PRV-infected neurons as we were unable to visualize NPY by IHC due to its rapid movement out of the cell body.

A large number of neurons in the paraventricular nucleus (PVN) were labelled by the PRV-GFP vectors (both Ba2001 and PRV^INTRO^). The PVN is an important integrative site for neuroendocrine and autonomic functions and contains two main divisions of neurons. Magnocellular neurons produce vasopressin or oxytocin and project to the posterior pituitary while parvocellular neurons produce CRH (± vasopressin) or TRH and project to the ARC/median eminence. In rodents, various stressors including inflammatory cytokines, hypoglycaemia, or physical restraint suppress both pulsatile LH release and the LH surge. Consequently, it has been suggested that CRH and/or vasopressin mediate the inhibitory effect of stressors on the HPG axis. Indeed, experimentally, both CRH and vasopressin inhibit LH secretion after central delivery in rodents [27, 28]. We did not find any PRV-GFP co-localization in vasopressin, oxytocin, cortocotropin releasing hormone (CRH) neurons in the PVN although around 56% of the GFP positive neurons were identified as expressing thyrotropin releasing hormone (TRH). The role that TRH might play in regulating the activity of *Kiss1* neurons is unknown but a direct action by this peptide is unlikely, as we have no data to support expression of the TRH receptor in *Kiss1* neurons from RNAseq or single cell RT-PCR analyses (unpublished). It is known however, that 90% of TRH neurons in the PVN are glutamatergic [29] and that the firing of ARC *Kiss1* neurons is increased in brain slices by exogenous glutamate agonists [30]. Thus, TRH neurons might provide glutamatergic inputs into ARC *Kiss1* neurons. The specific photostimulation protocol has been shown to induce release of glutamate from nerve terminals and result in c-FOS induction in targeted neurons [31]. However, light-activation of ChR2-expressing PVN neurons did not elicit either membrane current or c-FOS expression in ARC *Kiss1* neurons in this study although c-FOS activation in other neurons in the ARC was found indicating synaptic inputs from the PVN to the ARC.

There are several possible explanations for the lack of optogenetic responses in the ARC *Kiss1* neurons after ChR2 expression in the PVN. This may have been due to insufficient expression of ChR2 in the PVN although the c-FOS induction in non-*Kiss1* neurons in the ARC showed that we did manage to activate functional connections between these regions. Alternatively, the ChR2-expressing neurons were mainly located in the medial posterodorsal of the PVN, with very few neurons in the periventricular part of the PVN being transfected by the viral vector. Subsequently, the monosynaptic tracing showed that the primary afferents were from the PVNpe region while the PVNmpd region formed higher order inputs to the *Kiss1* neurons. It is challenging to target a specific neural population within a defined part of the PVN without the use of CRE-dependent transfection and the dispersed expression of ChR2 throughout the PVN may have contributed to the lack of response in the ARC *Kiss1* neurons. This would require more precise assessment via the appropriate CRE animals combined with a CRE-dependent AAV virus. These data highlight one of the limitations of using PRV vectors alone to undertake mapping studies–namely, the definitive identification of primary afferents and underline the importance of confirming circuits using different viral approaches.

It is therefore possible that the PVN-TRH neurons may influence *Kiss1* neurons indirectly, via another neuronal population in the ARC. Since the firing activity of TRH neurons is altered by signals of metabolic status such as α-MSH, NPY and leptin [32], this suggests that TRH neurons might communicate energy status to *Kiss1* neurons and thereby contribute to co-ordinating fertility with metabolism. The molecular identity of the remaining 44% of the PRV-GFP labelled neurons in the PVN has not yet been determined but could be neurons that sent autonomic outputs to the brain stem and spinal cord to regulate various aspects of energy metabolism. Alternatively, these neurons might express nNOS [33], neurotensin [34], cholecystokinin (CCK) [35] or dynorphin [36].

Viral spread to the ventral premammilary nucleus (PMV) is noteworthy since this region of the hypothalamus is a key site of action for leptin on fertility [37]. Leptin is produced by adipose tissue and provides a signal to the hypothalamus about peripheral energy stores and acts as a permissive signal for activation of the reproductive axis. Inactivation of the leptin receptor in *Kiss1* neurons does not affect mouse fertility indicating that the site of action of leptin is not directly on *Kiss1* neurons [37]. In contrast, expression of the leptin receptor specifically in the PMV region of LepR mutant mice restores fertility to these normally infertile mice [37]. This indicates that LepR expressing neurons in the PMV are important in relaying the leptin signal to the reproductive axis. In addition, it has recently been shown that glutamatergic PMV neurons that express the pituitary adenylate cyclase activating polypeptide (PACAP, *Adcyap1*) form monosynaptic connections with ARC *Kiss1* neurons and modulate reproductive function in female mice [38]. Whether the PRV-labelled neurons in the PMV express the leptin receptor and/or PACAP needs to be determined.

Neurons in the medial amygdala (MeA) form primary inputs into ARC *Kiss1* neurons. In rodents, the medial amygdala mediates olfactory stimuli such as pheromones [39] and modulates sexual behaviour [40]. Neurons in the amygdala are activated (c-FOS induction) by pheromones and anxiety. Significantly, intra-MeA injection of kisspeptin increases LH secretion in mice [41], alters male sexual behaviour [42], and pubertal timing [43]. Since both pheromones and anxiety can affect LH secretion and the reproductive axis, this suggests that the communication between the MeA and *Kiss1* neurons in the ARC is physiologically relevant.

The majority of the vasopressin neurons from the supraoptic nucleus (SON) formed synaptic connections with ARC *Kiss1* neurons. Although SON neurons have been reported to project mainly to the posterior pituitary [44], some anatomical studies have demonstrated projections of SON neurons to other brain regions, such as the PVN [45],[46]. Electrophysiological data in rats indicates that electrical stimulation of SON neurons results in activation of PVN neurosecretory cells [47]. Novel oxytocinergic signalling from the SON to ARC POMC neurons has also been reported [48]. Moreover, direct Neurokinin B (NKB) projections from the ARN to the SON of rats has been shown suggesting regulation of vasopressin release by NKB neurons, which also co-express kisspeptin [49]. Our data provide the first evidence that there is a reciprocal connectivity between the ARC *Kiss1* neurons and the SON vasopressin neurons. The physiological relevance of this projections remained to be investigated but a recent study indicates that arginine vasopressin activates intracellular calcium levels in the caudal region of *Kiss1* neurons in adult female mice [50].

*Kiss1* neurons in the rostral periventricular nucleus of the third ventricle (RP3V), which includes the AVPV, have been reported to send dense fibre projections to the ARC, raising the possibility of reciprocal connections between RP3V and ARC *Kiss1* neurons via other neurotransmitters [51]. Our data confirm that *Kiss1* neurons in the RP3V form synaptic connectivity with the ARC *Kiss1* neurons.

The secondary connections, which are only found in females, between the suprachiasmatic nucleus (SCN) and ARC *Kiss1* neurons is probably also relevant because the SCN is known to transmit information about day length to the reproductive axis, which is important in seasonal breeding [52]. In addition, a circadian signal from the SCN is required for the proestrous surge in GnRH/LH required for ovulation. The LH surge is controlled by *Kiss1* neurons in the AVPV region of the hypothalamus whose activity is gated by vasopressin signalling from the SCN in an estrogen-dependent manner [12, 17]. Our data suggest, that in addition to AVPV signalling, SCN neurons also signal to ARC *Kiss1* neurons, which may be important in reactivating the reproductive axis in seasonal breeders. However, our monosynaptic tracing data suggests that circadian signals derived from the SCN are indirect to the ARC *Kiss1* neurons. This is in agreement with a tracing study in rats using a combination of retrograde and viral tracers, which found no fibres originating from the SCN towards the ARC [53]. The connection from the SCN to the ARC is possibly mediated via the RP3V *Kiss1* neurons, which according to our data, are synaptically connected to the ARC *Kiss1* neurons.

The finding of inputs from the ventral tuberomammillary nucleus (VTM) raises the intriguing possibility that histaminergic neurons modulating the sleep-wake cycle may regulate the activity of ARC *Kiss1* neurons. This data is consistent with the observation that GnRH/LH pulsatility is directly altered by the sleep/wake state during the early follicular phase of women [54]. A study in rats revealed that subpopulations of histaminergic neurons innervate neurons in the caudal part of the ARC [55].

The subfornical organ (SFO) is one of the sensory circumventricular organs that is mainly involved in cardiovascular and neuroendocrine regulation [56, 57]. Metabolic signals like adiponectin, amylin, glucose, ghrelin, CCK and leptin have been shown to influence the activity of SFO neurons [58]. Although CCK is well established to inhibit food intake via its effects on the vagus nerve, CCK actions have been observed in SFO neurons [59, 60]. A retrograde tracing study in rats revealed that SFO neurons project directly to the ARN [61], suggesting the possible roles of SFO neurons in assimilating the peripheral signals from ghrelin and CCK, and relaying this signals to the ARC neurons.

Neurons in the interpeduncular nucleus (IPN) have been shown to receive inputs from the medial habenula, bed nucleus of stria terminalis and amygdala, brain regions that are mainly involved in emotion processing related to anxiety and fear. Substance P, a member of the tachykinin family of neuropeptides, play a major role in anxiety and depression [62] and has been shown to activate arcuate kisspeptin neurons as well as stimulate LH release [63]. The main source of Substance P is unknown and given that Substance P is found in the IPN of mice and rats [64, 65], this raises an interesting question of the role this IPN neurons in pathophysiology of anxiety and depression and their effects on the reproductive axis.

Our monosynaptic tracing results indicate that the temporal-spread of PRV does not accurately reflect the regions of the primary and secondary afferents of ARC *Kiss1* neurons. Indeed, studies shown that PRV spread is affected by the distance between the viral injection site and the afferent region, as well as the afferent terminal field density, where dense inputs can lead to faster spread compared to sparse innervations [66]. Given that our monosynaptic tracing experiment was performed only in adult males, it is important to undertake further assessments in adult or pre-pubertal female mice. Understanding the changes in these neuronal inputs under various physiological conditions in relation to the stimulation or inhibition of LH pulses is an important question to be resolved in the future.

## Conclusions

Our data provide a neuroanatomical description of populations of neurons that form synaptic inputs into *Kiss1* neurons in the arcuate as well as additional populations that form secondary or tertiary connections. We have identified inputs to ARC *Kiss1* neurons that may play a role in gating the activity of K*iss1* neurons with aspects of metabolism including POMC neurons and TRH neurons. This knowledge will be important in a functional analysis of these connections and how they regulate GnRH pulsatility at the level of ARC *Kiss1* neurons.

## Supporting information

S1 Fig PRV Bartha Ba2001 injections into *Kiss1*-Cre animals and wild-type animals. All wild-type animals did not show GFP-expressing cells in the ARC. ME, median eminence; 3V, 3^rd^ ventricle.
(DOCX)

S2 Fig Absence of co-localization between PRV-GFP and neuronal sub-types in the PVN region of the hypothalamus. Following PRV Bartha Ba2001 spread from ARC *Kiss1* neurons, immunohistochemistry was performed on brain sections to identify neuropeptides (red) that co-localized with GFP (green). CRH, corticotropin releasing hormone.
(DOCX)
