# Supplementary material for: Mapping neuronal inputs to Kiss1 neurons in the arcuate nucleus of the mouse
Source: PLoS One. 2019 Mar 27;14(3):e0213927. doi: 10.1371/journal.pone.0213927 (PMC6436706; doi:10.1371/journal.pone.0213927)
Supplement: S1 Fig — All wild-type animals did not show GFP-expressing cells in the ARC. ME, median eminence; 3V, 3rd ventricle. (DOCX) [file pone.0213927.s001.docx]

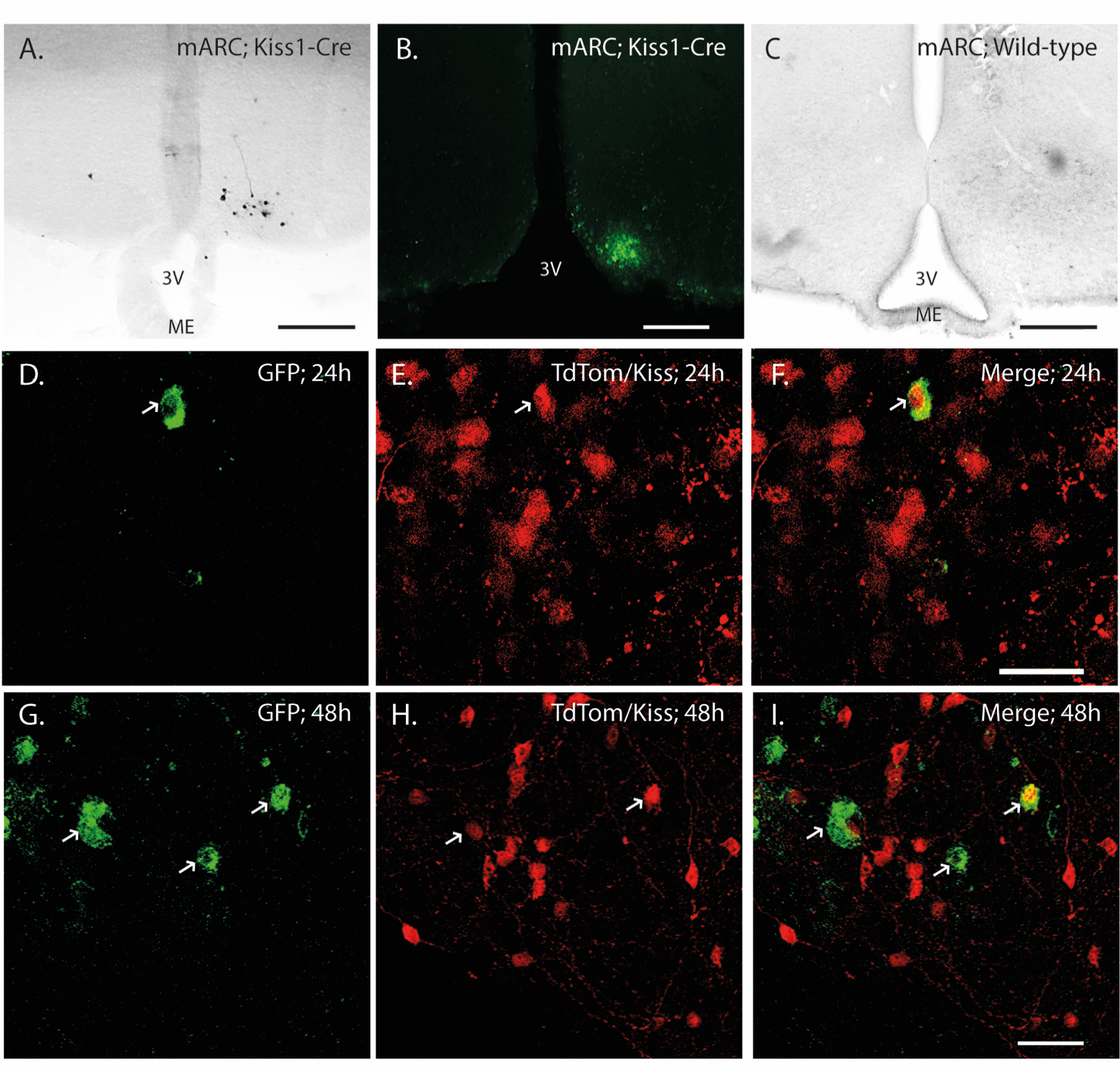


200 μM

**S1 Fig. PRV Bartha Ba2001 injections into *Kiss1*-Cre animals and wild-type animals.** All wild-type animals did not show GFP-expressing cells in the ARC.

ME, median eminence; ­ 3V, 3^rd^ ventricle.
