# Supplementary material for: Mapping neuronal inputs to Kiss1 neurons in the arcuate nucleus of the mouse
Source: PLoS One. 2019 Mar 27;14(3):e0213927. doi: 10.1371/journal.pone.0213927 (PMC6436706; doi:10.1371/journal.pone.0213927)
Supplement: S2 Fig — Following PRV Bartha Ba2001 spread from ARC Kiss1 neurons, immunohistochemistry was performed on brain sections to identify neuropeptides (red) that co-localized with GFP (green). CRH, corticotropin releasing hormone. (DOCX) [file pone.0213927.s002.docx]

**GFP+CRH**


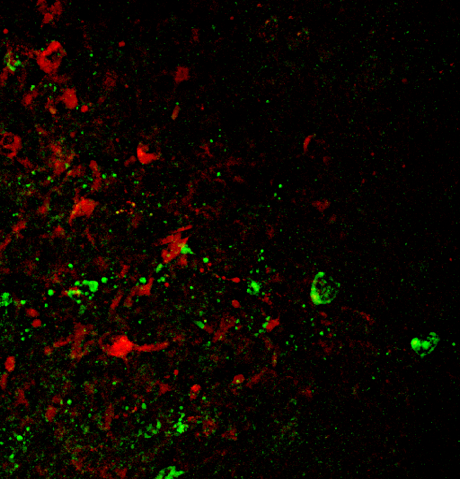


**GFP+Vasopressin**


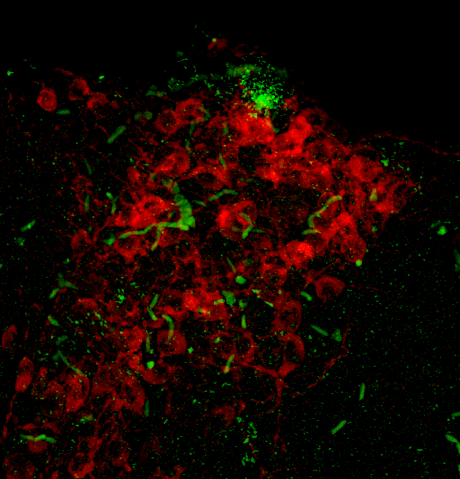


**45 μM**


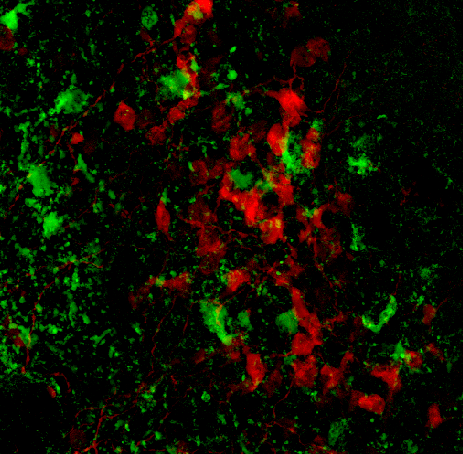


**GFP+Oxytocin**

**S2 Fig. Absence of co-localization between PRV-GFP and neuronal sub-types in the PVN region of the hypothalamus.**

Following PRV Bartha Ba2001 spread from ARC *Kiss1* neurons, immunohistochemistry was performed on brain sections to identify neuropeptides (red) that co-localized with GFP (green). CRH, corticotropin releasing hormone.
